# Effects of Different Thermal Treatments on Quality and Myofibrillar Protein Properties of Shelled Chinese Mitten Crab (*Eriocheir sinensis*) Meat

**DOI:** 10.3390/foods15193540

**Published:** 2026-10-04

**Authors:** Xihao Li, Changhu Xue, Zihao Wei

**Affiliations:** State Key Laboratory of Marine Food Processing & Safety Control, College of Food Science and Engineering, Ocean University of China, Qingdao 266404, China

**Keywords:** Chinese mitten crab, shell–meat separation, thermal treatment, myofibrillar protein

## Abstract

To reduce the difficulty of shell–meat separation while minimizing quality deterioration, this study compared the effects of two thermal treatments, traditional steaming (TS) and a part-specific mild steaming (MS) strategy, on the quality and myofibrillar protein (MP) properties of Chinese mitten crab meat, using raw crab meat (RM) as the reference. Results showed that the MS group exhibited pH and moisture content closer to those of RM, while no significant difference was observed in total volatile basic nitrogen and centrifugal loss compared with RM, suggesting that MS treatment caused less pronounced changes in physicochemical quality and water-holding properties. Meanwhile, electronic nose and electronic tongue analyses revealed that MS closely resembled RM, whereas TS deviated markedly, suggesting that MS treatment effectively preserved the natural sensory quality of crab meat. MS induced only moderate decreases in MP content, total and active sulfhydryl contents, and surface hydrophobicity. Its turbidity, particle size, and ultraviolet absorption spectra remained largely consistent with RM, while endogenous fluorescence and circular dichroism spectra exhibited substantially smaller changes than those in TS. These findings indicate that MS causes only mild conformational adjustments of MPs and less pronounced protein aggregation than TS. Overall, MS effectively reduces shell–meat separation difficulty while maximally preserving the inherent quality of crab meat.

## 1. Introduction

The Chinese mitten crab (*Eriocheir sinensis*), a unique aquatic economic species in China, is highly favored by consumers for its delicate meat and rich nutritional value. Its edible tissues are rich in high-quality proteins and essential amino acids, as well as unsaturated fatty acids and essential minerals, including zinc, iron, copper, and phosphorus, making it an important source of dietary nutrients [1]. In China, the annual production of Chinese mitten crab reaches approximately 0.89 million tons. The large production volume underscores its considerable economic importance to the freshwater aquaculture industry [2]. For a long time, the market circulation of this species has relied primarily on live sale. However, with the continuing expansion of aquaculture production in recent years, a large number of crabs have spoiled due to being unable to be sold in time, resulting in considerable economic losses. To alleviate the supply–demand imbalance of Chinese mitten crab and ensure a stable year-round supply of crab products, primary processing methods such as freezing and salting are currently widely adopted in production to extend the edible shelf life [3,4]. However, these processing methods yield relatively homogeneous products with low added value. In this context, implementing shell–meat separation of fresh crabs holds promise for diversifying the crab product line and further enhancing the economic value of crab products.

Raw crab meat that fully preserves the natural qualities of fresh meat serves as an ideal raw material for producing various crab meat products. However, because raw crab meat adheres tightly to the shell, direct shelling of fresh crabs is not only inefficient but also results in meat loss. To reduce the difficulty of shell removal, traditional processes usually subject whole crabs to steaming or boiling for a certain period. This thermal pretreatment is an important step in crab processing, as appropriate heating can facilitate shell–meat separation and improve processing safety, but excessive heating during traditional steaming may adversely affect the quality of crab meat [5]. To avoid the adverse effects of traditional heating, various approaches have been investigated. Among them, ultra-high pressure, a non-thermal processing technique, has become a research hotspot in the field of shell-removal processing for aquatic products such as crabs, shrimps, and shellfish [6,7]. However, owing to the high equipment cost and limited processing capacity, the widespread application of ultra-high pressure technology in the food industry still faces certain challenges [8]. Other shell-removal techniques are mostly confined to the laboratory scale and remain difficult to apply directly in the food industry [9,10]. Therefore, there is an urgent need to develop a shelling method that can balance crab meat quality with the practical demands of industrial production.

When processing meat separated from the shell, heat acts directly on the tissue. Therefore, to minimize the effects of secondary heating on shelled crab meat, controlling the temperature or heating time during shell–meat separation is crucial for maintaining the quality of ready-to-eat or processed crab meat products [11]. In traditional shell removal methods, excessively long heating times are a major factor affecting crab meat quality. If the steaming time is controlled so that the proteins at the shell–meat junction reach a degree of denaturation suitable for shell removal, rather than undergoing extensive and severe denaturation, the adverse effects of heat treatment on crab meat quality can be effectively minimized [12]. In addition, since the crab body is enclosed within the visceral tissue, heat must travel a relatively long distance to reach the shell–meat junction. In contrast, the shell–meat junctions on the crab legs and claws are directly exposed to steam, so they reach the appropriate level of doneness for shelling before the crab body does during the heat treatment process [13]. Based on this, the various parts of the crabs can be divided in advance, and mild heat treatment can be carried out for each part by adjusting the steaming time, to maximize the preservation of natural quality while avoiding modifications to the food processing plant’s existing shelling equipment.

Myofibrillar proteins (MPs), particularly myosin and actin, are a key muscle components directly linked to meat properties. As the major structural proteins of muscle fibers, myosin and actin are closely associated with muscle structure and water retention, thereby influencing the physicochemical and structural properties of crab meat [14]. Heat treatment can induce protein unfolding, conformational changes, and aggregation, thereby altering protein structure and functionality and affecting water retention and potentially the textural properties of crab meat. Appropriate heat treatment can foster a desirable protein network, whereas excessive heat may lead to severe protein aggregation, moisture loss, and toughening or overall quality degradation [15]. Therefore, understanding heat-induced changes in MP, particularly the denaturation and aggregation of myosin and actin, is important for elucidating the relationship between thermal processing and crab meat quality.

Different thermal processing methods can produce distinct quality outcomes in Chinese mitten crab, with heating mode and duration affecting the taste and metabolite profiles of crab muscle [1]. However, most studies have focused on the effects of cooking methods or alternative technologies on the quality of crab tissues, while thermal treatment specifically designed for shell–meat separation has received limited attention [6]. In particular, thermal treatment studies based on the different heat-transfer characteristics of crab legs, claws, and body remain limited.

**Figure 1:**
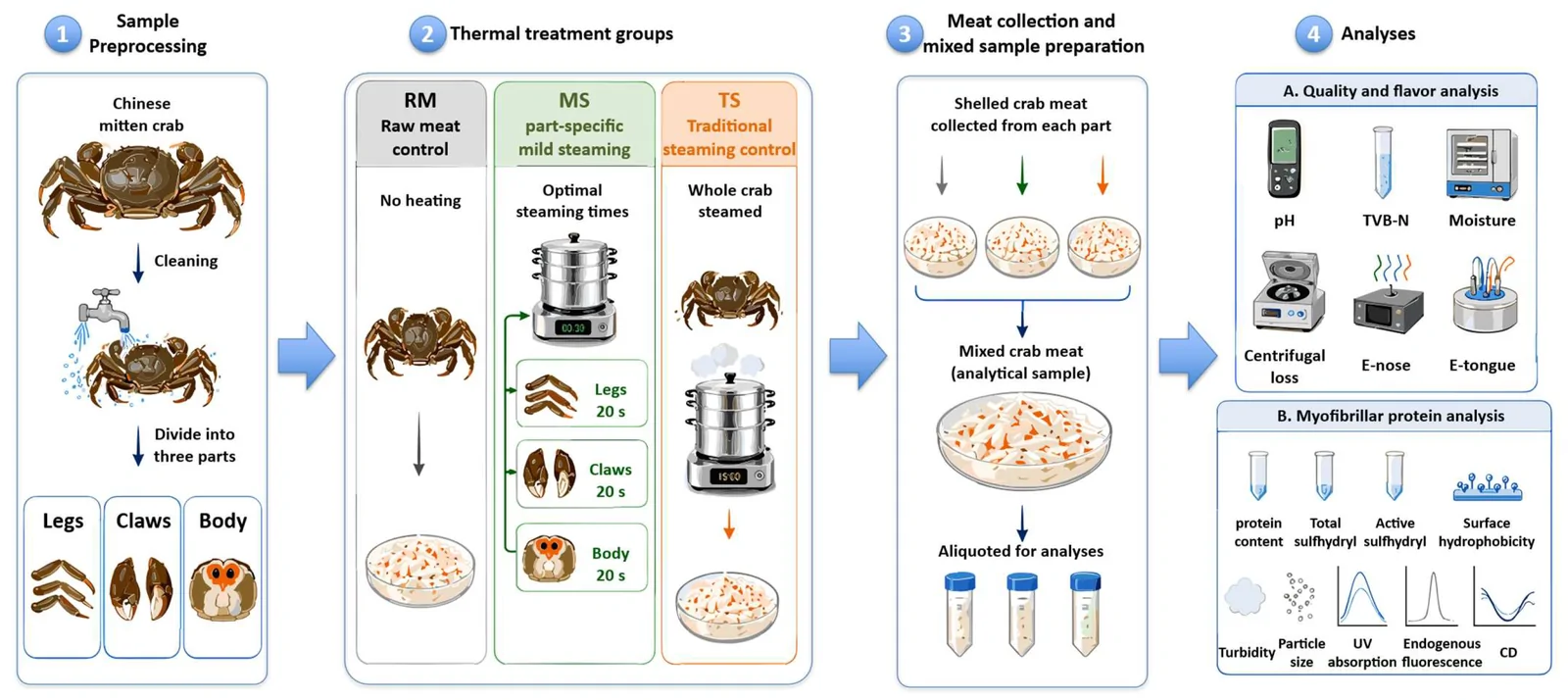
Flowchart illustrating the experimental design of this study.

The flowchart illustrating the experimental design of this study is shown in Figure 1. According to our previous study, the highest meat yield for crab legs, claws, and body was achieved at steaming times of 20 s, 20 s, and 60 s, respectively [16]. These optimized steaming conditions facilitated shell–meat separation and improved meat recovery. Based on these findings, this study further evaluated the physicochemical, sensory, and MP properties of the mixed crab meat to systematically investigate the effects of different thermal treatments on the quality of shelled meat from Chinese mitten crab. Through systematic evaluation from both macroscopic quality and molecular mechanism perspectives, this study aimed to further elucidate the effects of MS on crab meat quality and provide further theoretical support for its application in shell-removal processing in the crab processing industry.

## 2. Materials and Methods

### 2.1. Chemicals and Reagents

Potassium hydroxide (KOH), potassium chloride (KCl), hydrochloric acid (HCl), phosphoric acid (H_3_PO_4_, guaranteed reagent), and disodium ethylenediaminetetraacetate dihydrate (EDTA-2Na·2H_2_O) were purchased from Sinopharm Chemical Reagents Co., Ltd. (Shanghai, China). Boric acid (H_3_BO_3_), methyl red (biotechnology grade), magnesium oxide (MgO, 98% purity), tartaric acid (C_4_H_6_O_6_, ≥95% purity), and urea (CO(NH_2_)_2_) were purchased from Shanghai Macklin Biochemical Co., Ltd. (Shanghai, China). Bromocresol Green (indicator grade), tris(hydroxymethyl)aminomethane maleate (Tris–maleate, ≥99% purity), and sodium dodecyl sulfate (SDS, biotechnology grade) were purchased from Shanghai Yuanye Bio-Technology Co., Ltd. (Shanghai, China). Coomassie Brilliant Blue G-250 and 5,5′-dithiobis (2-nitrobenzoic acid) (DTNB, ≥98% purity) were purchased from Shanghai Aladdin Biochemical Technology Co., Ltd. (Shanghai, China). Bovine serum albumin (BSA, UV purity ≥98%) and tris(hydroxymethyl)aminomethane (Tris, ≥99.9% purity) were purchased from Beijing Solarbio Science & Technology Co., Ltd. (Beijing, China). Bromophenol Blue (BPB, ACS grade) was purchased from Jin Clon (Beijing) Biotechnology Co., Ltd. (Beijing, China). Anhydrous ethanol (C_2_H_5_OH) was purchased from Tianjin Fu Yu Fine Chemical Co., Ltd. (Tianjin, China). Unless otherwise specified, all other reagents were of analytical grade.

### 2.2. Material and Thermal Treatments

#### 2.2.1. Material

Live Chinese mitten crabs (males) were purchased from a local seafood market in Qingdao. The average crab weight was 116.63 ± 6.48 g. After purchase, they were tied and transported to the laboratory on ice and maintained alive in clean water at 4 °C. Before processing, they were washed and drained, and euthanized by heart piercing. The ARRIVE guidelines are provided in the Supplementary Materials.

#### 2.2.2. Thermal Treatments

For the raw crab meat (RM) group, fresh crabs received no heat treatment. Meat was manually harvested from the legs, claws, and body. The processing yield and shell–meat separation time were 11.03 ± 0.39% and 31.68 ± 2.96 min, respectively. The pooled meat was then minced and mixed using a meat grinder.

For the part-specific mild steaming (MS) group, fresh crabs were divided into three parts: legs, claws, and body. The carapace, gills, gonads, and remaining internal organs were sequentially removed from the body by hand, followed by thorough rinsing. The legs, claws, and body were steamed at 101.3 °C using a multifunctional electric steamer (ZN23FK530, Zhejiang Supor Co., Ltd., Taizhou, China) for 20 s, 20 s, and 60 s, respectively. Immediately after steaming, the final core temperatures of legs, claws, and body were measured as 54.8 ± 0.7 °C, 48.0 ± 0.6 °C, and 54.2 ± 2.6 °C, respectively. After cooling to 4 °C, meat was manually harvested from each part. The processing yield and shell–meat separation time were 22.20 ± 0.74% and 21.08 ± 0.36 min, respectively. The pooled meat was then minced and mixed using a meat grinder.

For the traditional steaming (TS) group, fresh crabs were steamed whole for 20 min at 101.3 °C using the multifunctional electric steamer (ZN23FK530). Immediately after steaming, the final core temperature of crabs was measured as 87.9 ± 2.7 °C. After cooling to 4 °C, meat was manually harvested from the legs, claws, and body. The processing yield and shell–meat separation time were 19.48 ± 0.37% and 18.13 ± 0.27 min, respectively. The pooled meat was then minced and mixed using a meat grinder.

The processing yield was calculated as the ratio of crab meat weight after shell–meat separation to the initial whole crab weight and expressed as a percentage. The shell–meat separation time was recorded using a stopwatch, from the beginning of manual separation to complete removal of edible meat from the shell. The final core temperature was measured immediately after steaming using an insertion thermometer (YZ-1650, China Yangzi Group Co., Ltd., Chuzhou, China), and the reading was recorded after stabilization.

pH, moisture content, centrifugal loss, electronic nose (E-nose), and electronic tongue (E-tongue) were measured immediately after sample preparation, whereas the remaining indicators were measured using crab meat stored at −80 °C.

### 2.3. pH

Crab meat (1.0 g) was homogenized in 10 mL of KCl (0.1 mol/L) using a homogenizer (FSH-2A, Changzhou Yuexin Instrument Manufacturing Co., Ltd., Changzhou, China) at 10,000 rpm for 30 s at room temperature. The pH was measured using a pH meter (ST3100, Ohaus, Parsippany, NJ, USA) [17].

### 2.4. Total Volatile Basic Nitrogen (TVB-N)

Crab meat (2.0 g, accurately weighed to 0.001 g) was mixed with 15 mL of distilled water and shaken vigorously until fully dispersed. After standing at room temperature for 30 min, the TVB-N content was determined using a fully automated Kjeldahl nitrogen analyzer (8400, FOSS Analytical AB, Höganäs, Sweden) [18].

### 2.5. Moisture Content

The moisture content of crab meat was determined according to the method described in AOAC (2000) [19]. Crab meat was placed in an oven (101-1AB-G, Beijing Zhongxingweiye Instrument Co., Ltd., Beijing, China) and dried at 103 °C until the difference between two consecutive weighings was less than 2 mg.

### 2.6. Centrifugal Loss

Crab meat (3.0 g) was placed in a tube and centrifuged at 12,000 rpm and 4 °C for 15 min (TGL20A, Changsha Yingtai Instrument Co., Ltd., Changsha, China). The supernatant was discarded and the remaining pellet was weighed. The centrifugal loss was calculated using the following formula [20].

(1) $$centrifugal\;loss=\frac{m_{0}-m_{1}}{m_{1}}\times 100$$

where *centrifugal loss* (%), and *m*_0_ and *m*_1_ represent the sample masses before and after centrifugation (g), respectively.

### 2.7. E-Nose

Crab meat (1.0 g) was sealed in a 10 mL headspace vial and equilibrated at room temperature for 60 min. The sample was then analyzed using an E-nose (PEN3, Airsense Analytics GmbH, Schwerin, Germany) with the following parameters: purified air as carrier gas at 400 mL/min, injection flow rate 400 mL/min, detection time 150 s, purge time 150 s, sampling interval 1 s [21].

### 2.8. E-Tongue

Crab meat (10.0 g) was homogenized with 20 mL of distilled water using a homogenizer (FSH-2A) at 10,000 rpm for 2 min at room temperature. The homogenate was then centrifuged (10,000 rpm, 4 °C, 10 min). The supernatant was filtered, and the pellet was re-extracted once under the same conditions. The combined filtrates were pooled, made up to 100 mL with distilled water, and transferred to an E-tongue sample cup for analysis (SA402B Plus-E, Intelligent Sensor Technology, Inc., Atsugi, Japan) [22].

The taste attributes evaluated by the E-tongue included sourness, bitterness, astringency, umami, saltiness, and aftertaste attributes. Aftertaste-A and Aftertaste-B represent the lingering sensations of astringency and bitterness, respectively.

### 2.9. MP Extraction and Quantification from Crab Meat

Crab meat (2.0 g) was mixed with 20 mL of ice-cold buffer 1 (50 mmol/L KCl, 20 mmol/L Tris-maleate, pH 7.0), homogenized using a homogenizer (FSH-2A) at 10,000 rpm for 60 s under ice-cold conditions, and centrifuged (10,000 rpm, 4 °C, 10 min). The pellet was re-extracted once under the same conditions, then suspended in 20 mL of ice-cold buffer 2 (0.6 mol/L KCl, 20 mmol/L Tris-maleate, pH 7.0), homogenized at 10,000 rpm for 60 s under ice-cold conditions, and incubated at 4 °C for 1 h. After centrifugation (10,000 rpm, 4 °C, 10 min), the supernatant was collected as the MP solution. Protein concentration was determined using the Bradford assay with BSA as the standard. The solution was stored at 4 °C, diluted to 1.0 mg/mL prior to measurement. All analyses were completed within 24 h [23].

### 2.10. Surface Hydrophobicity

An aliquot (500 μL) of protein solution was combined with 100 μL of BPB (1.0 mg/mL). After shaking for 15 min at room temperature, the mixture was centrifuged (10,000 rpm, 4 °C, 15 min). The supernatant was diluted 20-fold with buffer 2, and its absorbance was measured at 595 nm (UV2355, UNICO (Shanghai) Instrument Co., Ltd., Shanghai, China). Surface hydrophobicity was calculated using the following equation [24].

(2) $$\mathrm{HY}=\frac{\left(A_{1}-A_{2}\right)}{A_{1}}\times 200$$

where HY is surface hydrophobicity (μg/mg), and *A*_1_ and *A*_2_ are the absorbances of the blank and sample, respectively.

### 2.11. Total Sulfhydryl

An aliquot (0.5 mL) of protein solution was mixed with 4.5 mL Buffer A (48.048 g urea, 2.423 g Tris, 0.3722 g EDTA-Na_2_·2H_2_O, 2 g SDS, pH 7.0, made up to 100 mL), vortexed, and kept at room temperature for 30 min. A 4 mL aliquot was then combined with 0.4 mL DTNB (0.1 g DTNB, 2.423 g Tris, pH 8.0, made up to 100 mL) and incubated at 40 °C for 25 min. Absorbance was measured at 412 nm using a UV2355 spectrophotometer. Total sulfhydryl content was calculated using the following equation [25].

(3) $$X_{1}=\frac{A\times D}{\varepsilon \times C_{0}}\times 10^{6}$$

where X_1_ is the total sulfhydryl content (μmol/g), *C*_0_ is the mass concentration of MPs (mg/mL), *A* is the absorbance at 412 nm, *D* is the dilution factor, and *ε* is the molar extinction coefficient, which was set at 13,600 L/(mol·cm).

### 2.12. Active Sulfhydryl

Active sulfhydryl content was measured using the same procedure as that for total sulfhydryl content, except that Buffer A was prepared without urea [25].

### 2.13. Turbidity

The absorbance of the protein solution at 600 nm was measured using a UV2355 spectrophotometer as an indicator of turbidity [26].

### 2.14. Particle Size

Particle size was determined using a nanoparticle size analyzer (ZS90, Malvern Panalytical Ltd., Malvern, UK) after equilibration for 120 s [27].

### 2.15. Ultraviolet Absorption (UV) Spectra

The UV spectrum was measured using a UV2355 spectrophotometer from 190 to 400 nm at 1 nm intervals [28].

### 2.16. Endogenous Fluorescence Spectra

The endogenous fluorescence spectrum was measured using a fluorescence spectrophotometer (F-4600, Hitachi, Ltd., Tokyo, Japan) under the following conditions: excitation wavelength of 280 nm, emission wavelength range of 280–400 nm, scan speed of 240 nm/min, and excitation and emission slit widths of 2.5 nm [29].

### 2.17. Circular Dichroism (CD) Spectra

The protein solution and buffer 2 were diluted 20-fold with deionized water. CD spectra were recorded using a CD spectrometer (Jasco-1500, JASCO Corporation, Tokyo, Japan) under the following conditions: wavelength range of 190–260 nm, scan speed of 100 nm/min, D.I.T. of 2 s, and bandwidth of 1 nm. The relative secondary structure contents were estimated using the Secondary Structure Estimation database [30].

### 2.18. Statistical Analysis

All data were expressed as mean ± standard deviation (*n* = 3), where *n* = 3 represents three parallel measurements of the same pooled sample. Origin 2024 software was used for graph construction. Statistical analysis was performed using SPSS 27.0 with one-way ANOVA, *p* < 0.05 was considered statistically significant. The flowchart was prepared using Microsoft PowerPoint.

## 3. Results and Discussion

### 3.1. pH and TVB-N

**Figure 2:**
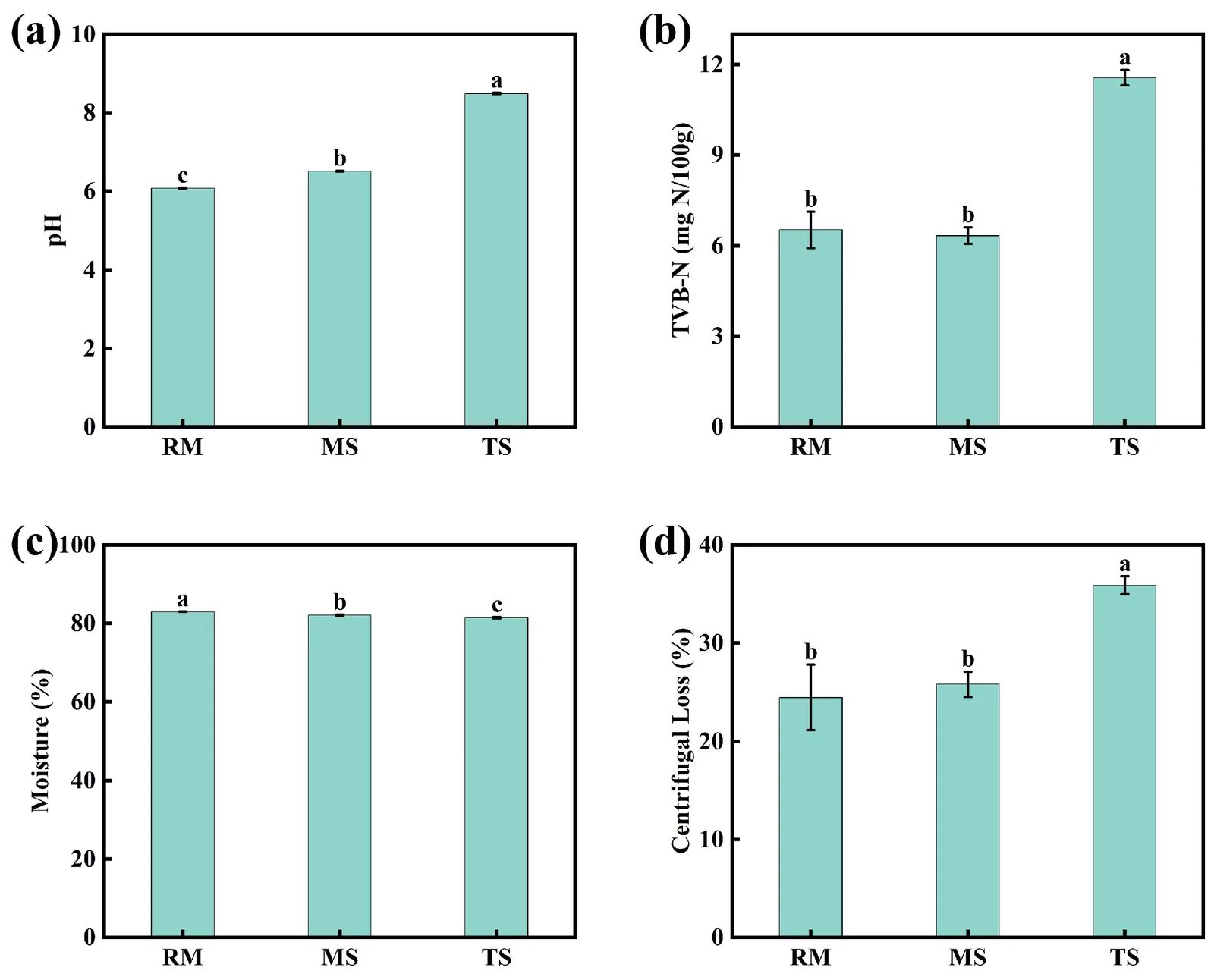
Physicochemical properties of crab meat under different thermal treatments. (**a**) pH; (**b**) total volatile basic nitrogen; (**c**) moisture content; (**d**) centrifugal loss. RM, raw meat; MS, mild steaming; TS, traditional steaming. Different letters indicate significant differences among groups (*p* < 0.05).

pH and TVB-N are two key parameters used to characterize changes in the physicochemical properties of crab meat and the accumulation of alkaline nitrogenous compounds during thermal processing [31]. The pH and TVB-N values of crab meat under different thermal treatments are presented in Figure 2a,b. The pH of the RM group was 6.08 ± 0.01, the pH in the MS group increased slightly (6.51 ± 0.01), while the pH in the TS group exhibited a significant increase to 8.49 ± 0.02 (*p* < 0.05). No significant difference in TVB-N content was found between the RM and MS groups, while the TS group exhibited a marked increase to 11.5 mg N/100 g (*p* < 0.05).

The above changes may result from different heating conditions and their effects on protein structure. A short steam treatment of the MS group may have disrupted some hydrogen bonds and hydrophobic interactions in the MPs, causing moderate protein denaturation. Structural changes may have exposed some buried basic amino acid residues and increased the proton-binding capacity of the protein, thus causing a small rise in pH [24]. In addition, the absence of a significant change in TVB-N content suggests that no substantial accumulation of volatile basic nitrogenous compounds occurred under MS treatment [32].

In contrast, prolonged heating in the TS group not only induced conformational changes in the crab meat proteins but also may have promoted thermal degradation, thereby releasing more free amino acids and small nitrogen-containing compounds. As a result, both pH and TVB-N increased substantially [33]. Moreover, the pH rise observed in the TS group could be partly attributed to the potential contribution of alkaline mineral components derived from the crab shells during prolonged steaming [34].

### 3.2. Moisture Content

The moisture content of crab meat under different thermal treatments is presented in Figure 2c. The moisture content in the RM group was 83.01 ± 0.06%, whereas those in the MS and TS groups decreased to 82.09 ± 0.12% and 81.47 ± 0.17%, respectively. Compared with the TS group, the MS group retained 0.62 percentage points more moisture, which may have practical economic implications in large-scale processing.

The variations in moisture content mainly reflected the extent of muscle fiber contraction caused by heating intensity. In the TS group, prolonged heating may have induced more extensive denaturation and aggregation of MPs, which in turn caused pronounced longitudinal contraction of muscle fibers. As a result, the spacing between myofibrillar lattices was markedly reduced, and the water retained within the myofibrillar structure was expelled from the lattice structure and migrated to the extracellular spaces, ultimately being lost as cooking drip [35]. In contrast, in the MS group, heat treatment induced only moderate conformational adjustments of MPs, and the native conformation of muscle fibers was largely retained. The myofibrillar lattice may have undergone less extensive structural contraction, thereby allowing more effective moisture retention.

### 3.3. Centrifugal Loss

Centrifugal loss reflects the extent of water loss from a protein’s three-dimensional network under external mechanical forces. The centrifugal loss of crab meat subjected to different thermal treatments is presented in Figure 2d. No significant difference in centrifugal loss was observed between the RM group (24.47 ± 3.33%) and the MS group (25.80 ± 1.29%). However, the centrifugal loss of the TS group (35.89 ± 0.91%) was significantly higher (*p* < 0.05).

The variation in centrifugal loss may be attributed to differences in the extent of protein network disruption under different thermal treatment conditions. Compared with TS, the MS group exhibited less pronounced changes in MP structure, and its three-dimensional protein network was relatively well preserved, thereby maintaining better water-holding capacity during centrifugation [36]. In contrast, the extended heating applied to the TS group promoted more extensive protein aggregation and intermolecular interactions, which may have disrupted the protein network and resulted in greater water release during centrifugation [37].

The water-holding capacity of meat is closely associated with its textural properties, as heat-induced protein denaturation and structural changes can alter water retention and texture [38]. Considering the moisture content and centrifugal loss, the MS group exhibited better water retention than the TS group, which may favor a softer and juicier texture. In contrast, the greater moisture loss and centrifugal loss in the TS group may be associated with a firmer and less juicy texture under prolonged heating [39].

### 3.4. E-Nose

**Figure 3:**
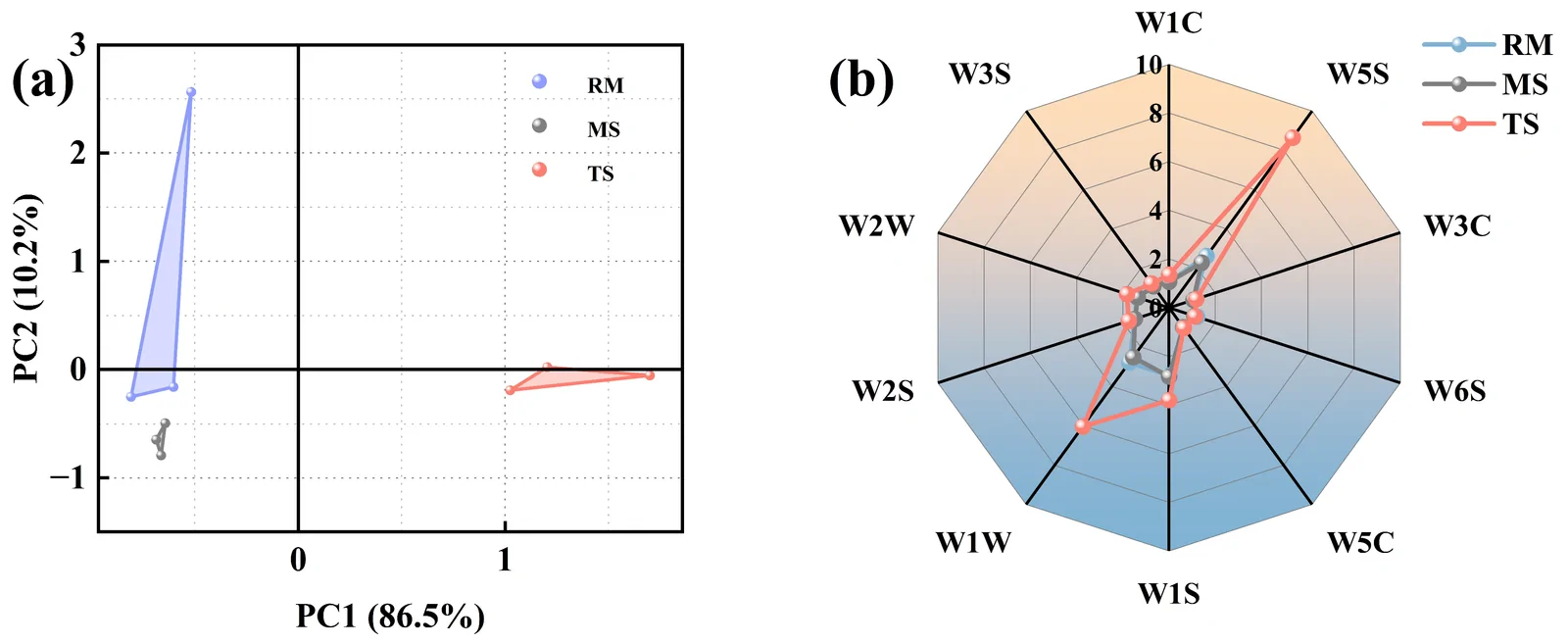
(**a**) PCA score plot of electronic nose data for crab meat subjected to different thermal treatments. (**b**) Radar chart of electronic nose sensor responses. RM, raw meat; MS, mild steaming; TS, traditional steaming. The PEN3 electronic nose sensor array consisted of 10 sensors: W1C (sensitive to aromatic compounds), W5S (sensitive to nitrogen oxides), W3C (sensitive to ammonia and aromatic compounds), W6S (sensitive to hydrogen), W5C (sensitive to alkanes and aromatic compounds), W1S (sensitive to methane), W1W (sensitive to sulfides), W2S (sensitive to ethanol), W2W (sensitive to aromatic compounds and organic sulfides), and W3S (sensitive to alkanes).

The E-nose emulates the odor-recognition behavior of the mammalian olfactory system to detect volatile flavor characteristics in a sample. Principal Component Analysis (PCA) of crab meat after different thermal treatments is shown in Figure 3a. PC1 and PC2 together explained 96.7% of the total variance, and their individual contributions were 86.5% and 10.2%, respectively. These two components accounted for most of the variation in the volatile flavor characteristics of the samples. Notably, the MS group and the RM group were closely positioned, suggesting that mild steaming caused relatively limited changes in the overall volatile flavor profile. On the other hand, the TS group was far from the RM and MS groups, indicating that prolonged steaming led to a significant change in the volatile flavor characteristics of the crab meat.

The radar chart of the E-nose responses to different thermal treatments of crab meat is shown in Figure 3b. The sensor response profiles of the RM and MS groups closely overlapped, further confirming that mild steaming caused relatively limited changes in the original flavor of the crab meat. The TS group exhibited significantly elevated response values on the W5S (nitrogen oxides) and W1W (sulfides) sensors. The enhanced W5S response may be due to the thermal degradation of trimethylamine oxide into trimethylamine and other volatile amines under extended heating [40]. Similarly, the increased W1W response is likely ascribed to the heat-induced breakdown of sulfur-containing amino acids, leading to the formation of volatile sulfur compounds such as methyl mercaptan and dimethyl sulfide [41].

### 3.5. E-Tongue

**Figure 4:**
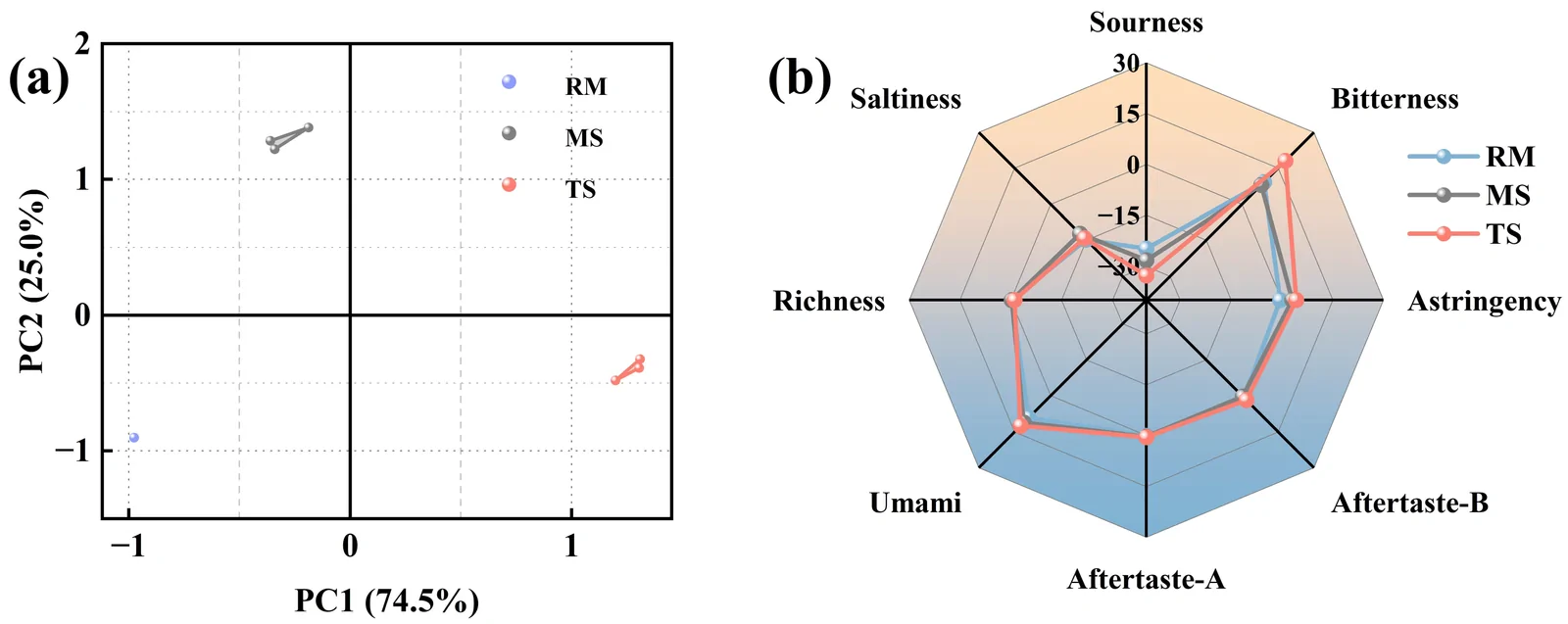
(**a**) PCA score plot of electronic tongue data for crab meat subjected to different thermal treatments. (**b**) Radar chart of electronic tongue sensor responses. RM, raw meat; MS, mild steaming; TS, traditional steaming.

The E-tongue simulates the human taste perception mechanism, enabling rapid differentiation and evaluation of the taste profiles of different samples. PCA results for crab meat subjected to different thermal treatments are presented in Figure 4a. A cumulative variance of 99.5% was obtained from PC1 (74.5%) and PC2 (25.0%), indicating that most of the taste variation among samples was captured by these two components. Specifically, the MS group and the RM group were relatively close along the PC1 axis, whereas the TS group deviated markedly in the positive direction of PC1, suggesting that the overall taste profile of the MS group was more similar to that of fresh crab meat.

The radar chart of the E-tongue responses for crab meat subjected to different thermal treatments is presented in Figure 4b. Compared with the RM group, the sourness response values of both the MS and TS groups were lower, with the decrease being more pronounced in the TS group. The main reason for this decrease may be the evaporation and thermal decomposition of organic acids during heating [42]. In addition, an alkaline environment in the system may change the ionization state of organic acids and thus alter the response of the sensor to sourness [43]. Both the MS and TS groups showed higher response values than the RM group for umami, and the TS group had a more pronounced increase. The increase may be due to the heat-induced release of taste-active nucleotides and free amino acids from the crab meat [22].

For bitterness, the MS and RM groups showed relatively similar response values, whereas the TS group exhibited a marked increase. This increase is likely due to the extensive hydrolysis of peptide bonds induced by prolonged heating, resulting in the formation of bitter peptide fragments containing hydrophobic amino acid residues. Concurrently, the released hydrophobic amino acids themselves may also contribute to the bitter taste [44]. Collectively, these findings are consistent with the observed changes in the TVB-N data, suggesting that more extensive thermal treatment and associated protein degradation may be associated with the formation or accumulation of compounds contributing to bitter taste.

The physicochemical and sensory properties of crab meat were affected by the thermal treatment conditions. Compared with TS, the shorter heating duration in MS resulted in less extensive thermal exposure and milder changes in crab meat overall quality, with relatively stable pH and TVB-N levels, better moisture retention, lower centrifugal loss, and sensory characteristics closer to RM. In contrast, prolonged heating in TS caused more pronounced changes in physicochemical and sensory properties. These differences were primarily attributed to the effects of thermal treatment on crab meat protein structures. Therefore, the changes in MP properties were further investigated to elucidate the potential mechanisms underlying these quality differences.

### 3.6. MP Content

**Figure 5:**
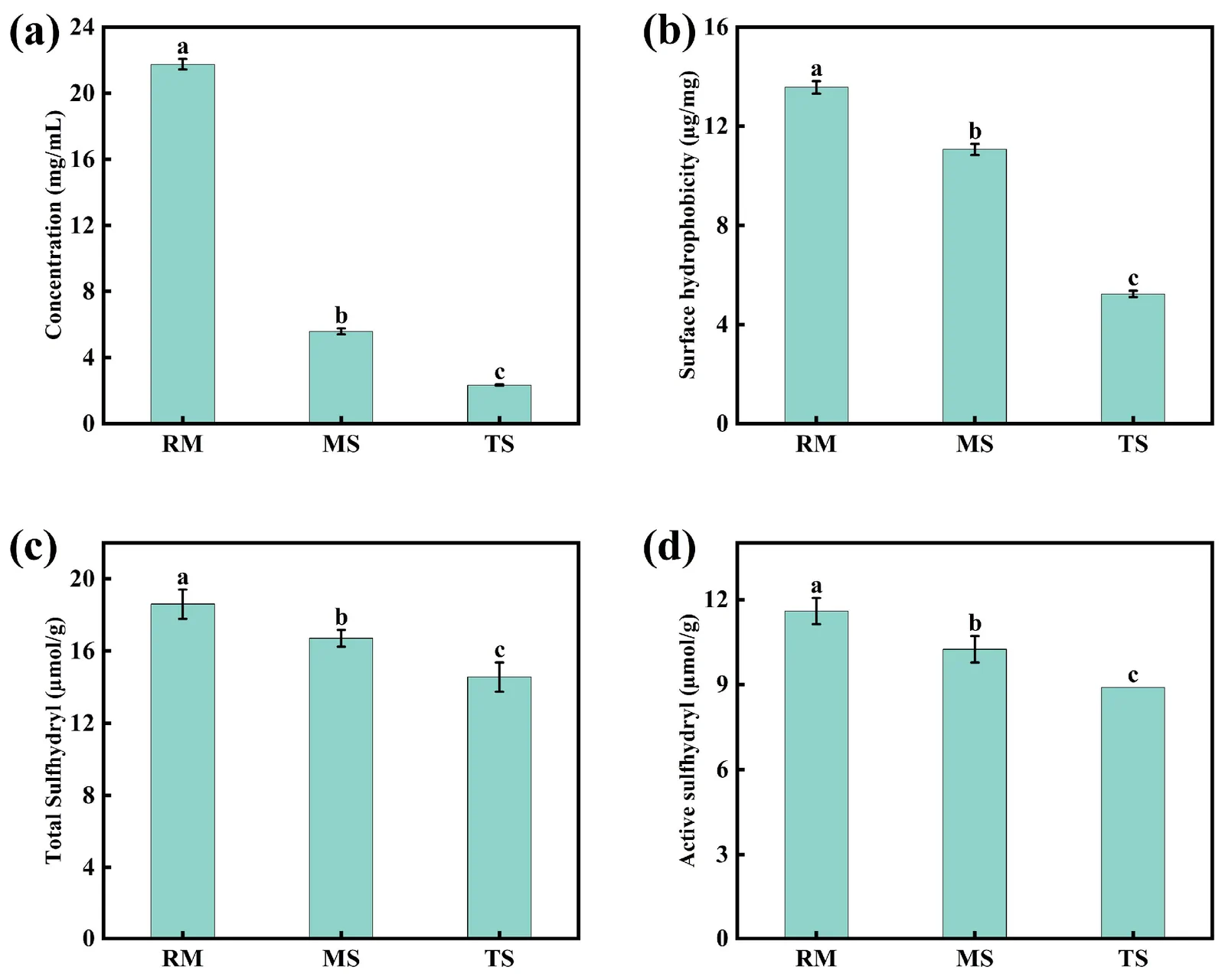
Biochemical characteristics of myofibrillar proteins (MPs) in crab meat subjected to different thermal treatments. (**a**) MP content; (**b**) surface hydrophobicity; (**c**) total sulfhydryl content; (**d**) active sulfhydryl content. RM, raw meat; MS, mild steaming; TS, traditional steaming. Different letters indicate significant differences among groups (*p* < 0.05).

The amounts of MPs in crab meat after various thermal treatments are shown in Figure 5a. The contents of the RM, MS, and TS groups were 21.75 ± 0.31 mg/mL, 5.58 ± 0.18 mg/mL and 2.35 ± 0.04 mg/mL, respectively. A significant decreasing trend was observed with increasing heat treatment intensity (*p* < 0.05).

The lower extractable MP content observed in the MS and TS groups may be related to heat-induced changes in MP structure. Myosin and actin are major MPs that contribute to muscle structure and meat quality. Myosin is relatively heat-sensitive and generally denatures at approximately 40–60 °C, whereas actin is more heat-stable and typically denatures at approximately 70–80 °C [45]. In the present study, the final core temperatures of the crab legs, claws, and body in the MS group were 54.8 ± 0.7 °C, 48.0 ± 0.6 °C, and 54.2 ± 2.6 °C, respectively, which were within the typical denaturation range of myosin but below that of actin. Thus, MS may have induced partial unfolding and denaturation of MPs. In contrast, the TS group reached a final core temperature of 87.9 ± 2.7 °C, exceeding the typical denaturation temperatures of both proteins and likely promoting more extensive denaturation and aggregation. Heat-induced unfolding and denaturation can disrupt protein structure and reduce protein solubility, thereby decreasing protein extractability [24].

### 3.7. Surface Hydrophobicity

The surface hydrophobicity of MPs extracted from crab meat subjected to different thermal treatments is presented in Figure 5b. The values for the RM, MS, and TS groups were 13.57 ± 0.26 μg/mg, 11.06 ± 0.22 μg/mg, and 5.24 ± 0.13 μg/mg, respectively. Compared with the RM group, the MS group showed a slight decrease, whereas the TS group exhibited a marked reduction (*p* < 0.05).

A reduction in the surface hydrophobicity was mainly caused by heat-induced denaturation of the protein and subsequent reaggregation. Thermal treatment induces the unfolding of myosin, actin, and other MPs by disrupting non-covalent interactions, exposing normally buried hydrophobic residues. These exposed hydrophobic groups can subsequently interact with hydrophobic regions of neighboring protein molecules, promoting intermolecular association and aggregation. As aggregation proceeds, some hydrophobic residues may become reburied within the aggregates, thereby reducing their accessibility to BPB and resulting in lower surface hydrophobicity [46]. The decrease in surface hydrophobicity in the MS group was less pronounced than that in the TS group, indicating that relatively limited conformational changes occurred following MS treatment.

### 3.8. Total Sulfhydryl and Active Sulfhydryl

Total sulfhydryl content reflects the extent of oxidative loss of sulfhydryl groups, whereas active sulfhydryl content is used to characterize the combined effects of conformational changes and oxidative denaturation [47]. The effects of different thermal treatments on the total and active sulfhydryl contents of MPs in crab meat are presented in Figure 5c,d. The total sulfhydryl contents in the RM, MS, and TS groups were 18.60 ± 0.81 μmol/g, 16.72 ± 0.47 μmol/g, and 14.56 ± 0.81 μmol/g, respectively. The active sulfhydryl contents in the corresponding groups were 11.59 ± 0.47 μmol/g, 10.24 ± 0.47 μmol/g, and 8.90 ± 0.00 μmol/g, respectively. The trends of these two datasets were highly consistent. The decrease in the MS group was relatively moderate, whereas the TS group exhibited a significantly greater reduction (*p* < 0.05).

The drop in sulfhydryl groups was likely associated with heat-induced oxidation. The oxidative damage caused by short-term heat treatment was relatively small compared with that induced by prolonged steaming [48]. Sulfhydryl groups embedded in the protein interior are indirectly reflected by the difference between total and active contents. The differences were 7.01 μmol/g in the RM group, 6.48 μmol/g in the MS group, and 5.66 μmol/g in the TS group, showing a progressive decrease with increasing heat treatment intensity. This observation suggests that heating induces conformational changes in myosin, actin, and other proteins, exposing previously buried sulfhydryl groups to the aqueous environment, where they may undergo oxidation to form disulfide bonds [49]. The TS group showed a smaller difference between total and active sulfhydryl contents, suggesting that prolonged high-temperature exposure caused more extensive protein denaturation and oxidative damage.

### 3.9. Turbidity

**Figure 6:**
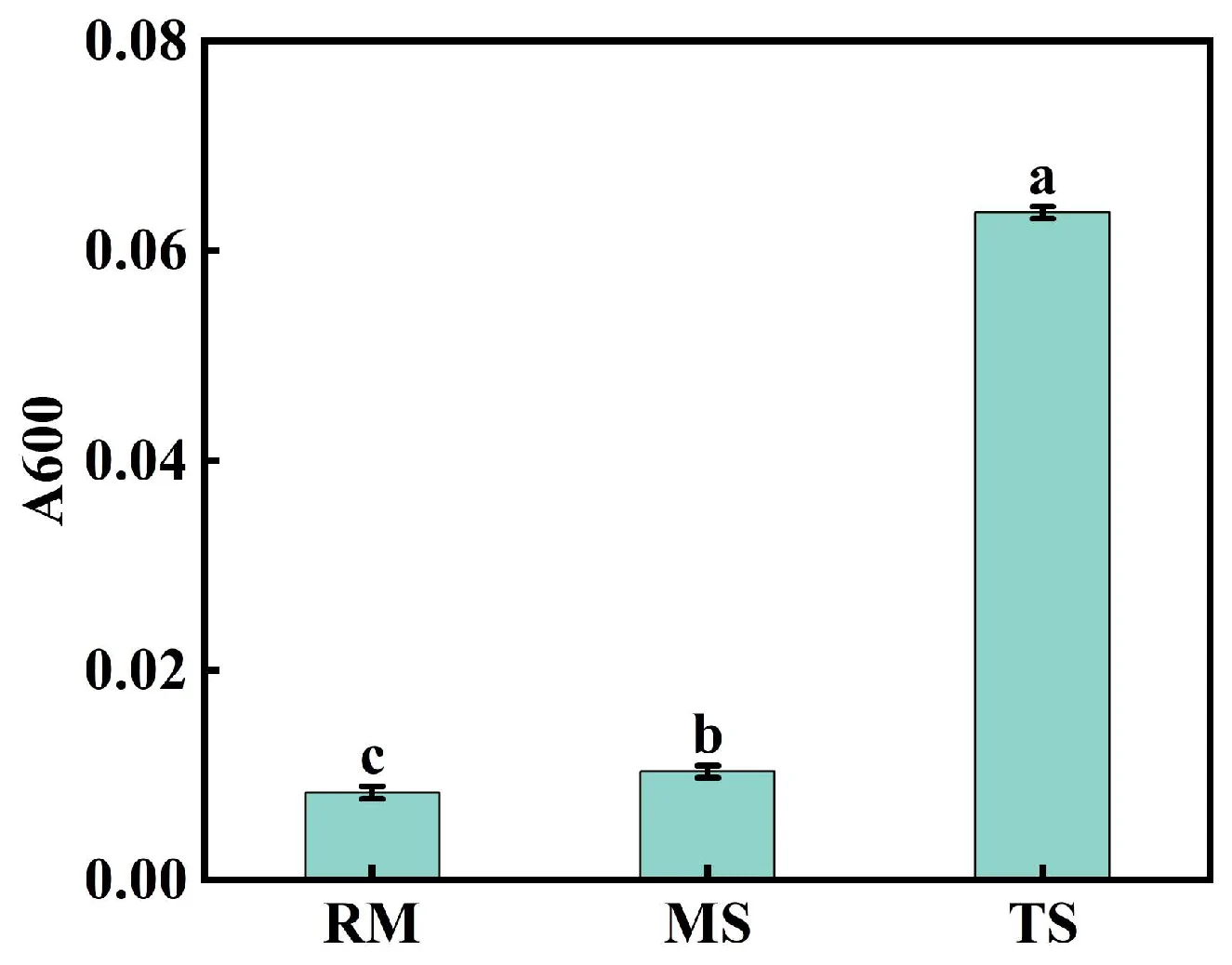
Turbidity of MPs extracted from crab meat subjected to different thermal treatments. RM, raw meat; MS, mild steaming; TS, traditional steaming. Different letters indicate significant differences among groups (*p* < 0.05).

The turbidity value is widely used as a measure of protein aggregation, with higher absorbance generally indicating the formation of larger particles. Turbidity values of MP extracts from crab meat after different thermal treatments are shown in Figure 6. The turbidity values of the RM and MS groups were virtually the same (0.008 ± 0.001 and 0.010 ± 0.001, respectively), whereas the TS group was significantly higher at 0.064 ± 0.001 (*p* < 0.05).

The turbidity results suggest that the MS treatment induced only limited protein denaturation, with the protein components in the supernatant remaining relatively well dispersed and producing limited light scattering. In contrast, the turbidity of the TS group increased markedly, indicating that extensive denaturation and protein aggregation induced by prolonged heating may have resulted in the formation of larger protein aggregates [50]. This result was corroborated by the surface hydrophobicity results. Heat-induced protein unfolding can expose previously buried groups, which may enhance intermolecular interactions and promote protein aggregation, thereby increasing turbidity.

### 3.10. Particle Size

**Figure 7:**
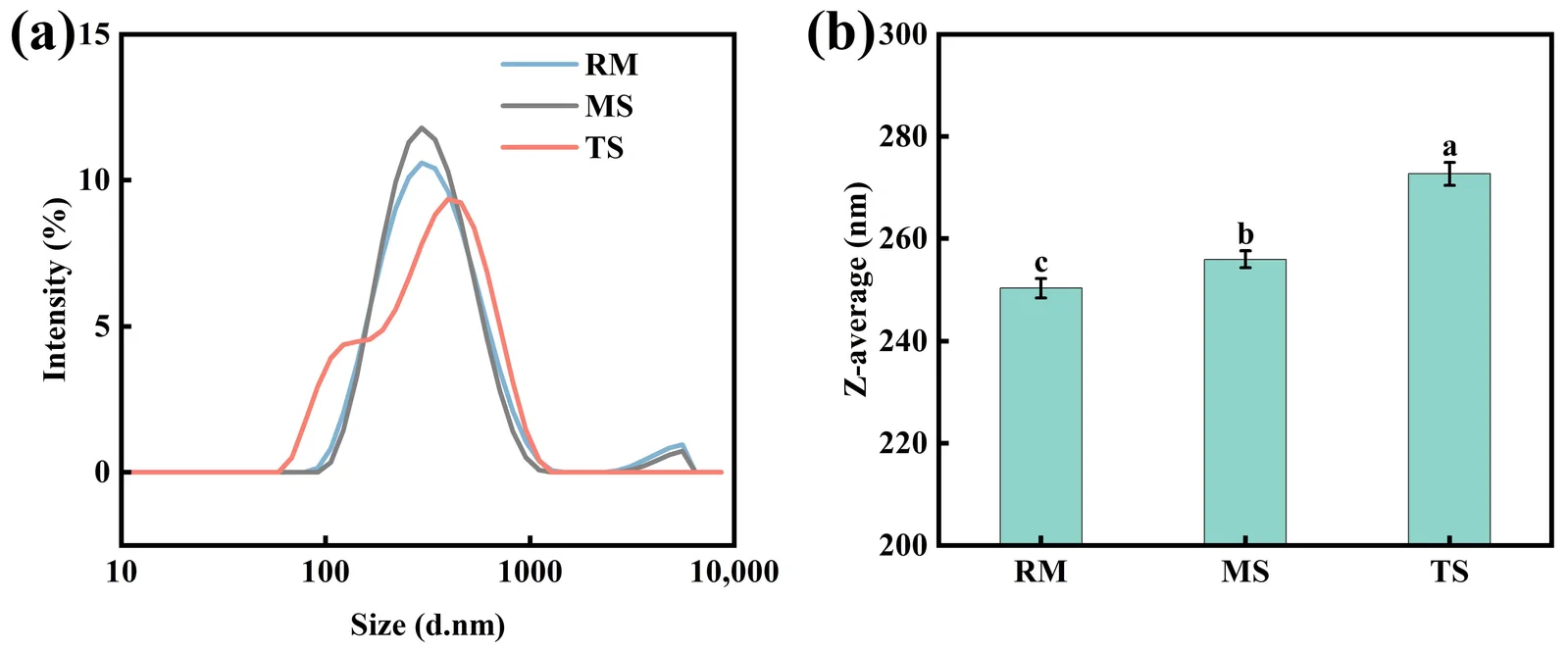
(**a**) Particle size distribution and (**b**) average particle size of MPs extracted from crab meat subjected to different thermal treatments. RM, raw meat; MS, mild steaming; TS, traditional steaming. Different letters indicate significant differences among groups (*p* < 0.05).

Particle size distribution (a) and average particle size (b) of MPs in crab meat after different thermal treatments are shown in Figure 7. The particle size distribution curves of the RM and MS groups were almost the same, both showing a single peak, and their average particle sizes were also close (250.27 ± 1.90 nm and 256.00 ± 1.64 nm, respectively), indicating relatively concentrated particle size ranges and the absence of pronounced protein aggregation. These findings are highly consistent with the turbidity data, further supporting the conclusion that the MS treatment caused only limited changes in protein conformation [51].

The TS group’s particle size distribution curve was much broader than that of the MS group and exhibited a multi-modal pattern. Furthermore, its average particle size increased significantly to 272.70 ± 2.26 nm (*p* < 0.05), indicating that protein particles of various sizes existed in the solution. This may be attributed to protein denaturation, aggregation, and intermolecular interactions caused by extended high-temperature treatment. Protein unfolding may expose reactive groups and promote intermolecular interactions, thereby facilitating protein aggregation and leading to an increase in the average particle size [52].

### 3.11. UV Spectra

**Figure 8:**
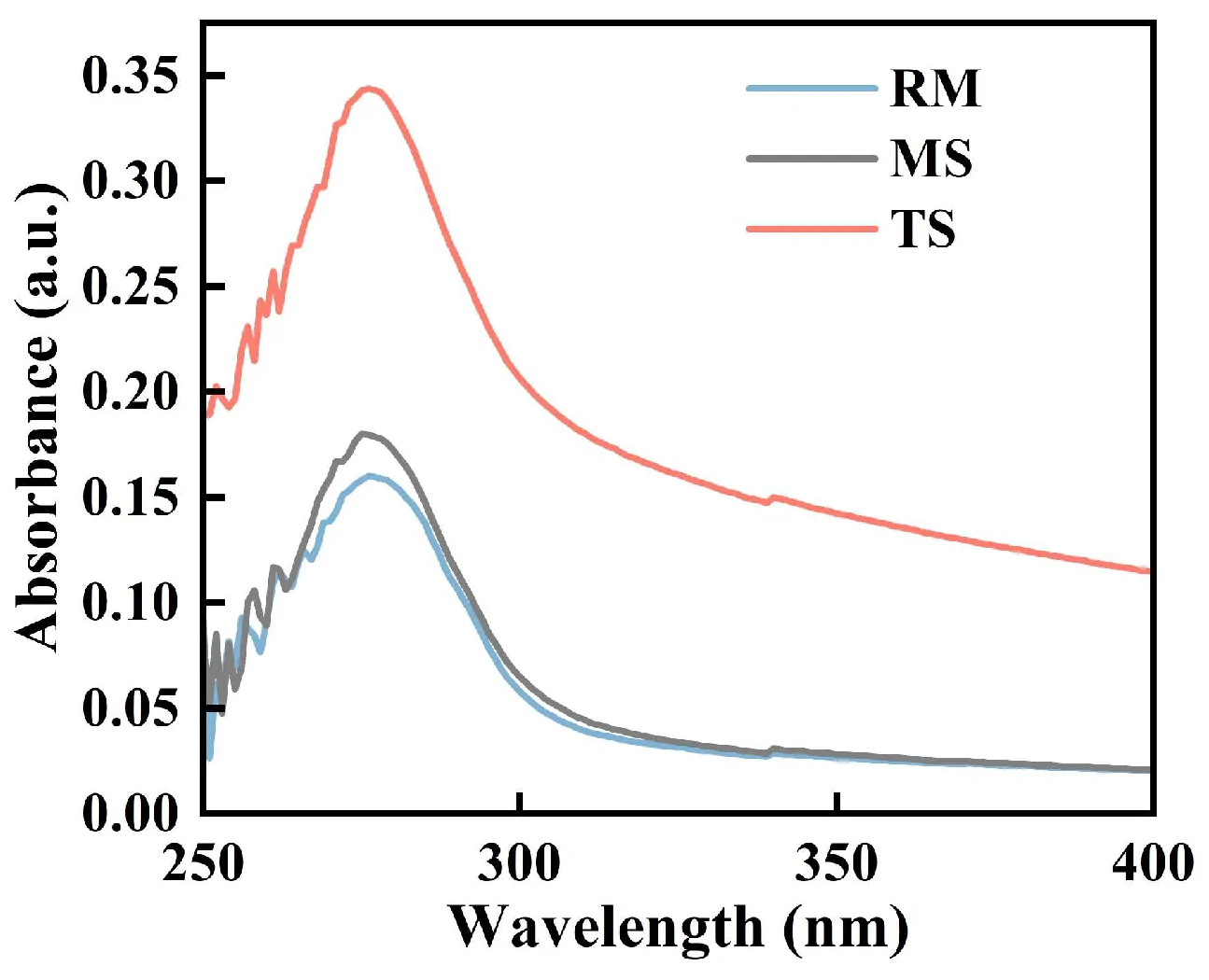
Ultraviolet absorption spectra of MPs extracted from crab meat subjected to different thermal treatments. RM, raw meat; MS, mild steaming; TS, traditional steaming.

The UV spectra of MPs in crab meat after different thermal treatments are shown in Figure 8. The spectra of the RM and MS groups were almost the same, except that the MS group showed a slight increase in absorbance, suggesting that brief heating did not significantly alter the local environment of the aromatic residues.

In the TS group, the ultraviolet absorption intensity was significantly higher than that of the RM and MS groups, which may be attributable to two factors. First, prolonged high-temperature treatment induced extensive protein denaturation, promoting the unfolding of MPs and exposing numerous originally buried aromatic amino acid residues to the polar solvent environment. This exposure may increase their molar extinction coefficients, thereby contributing to the elevated absorbance [53]. Second, free aromatic amino acids and small peptide fragments that may be generated from thermal protein degradation also absorb in the ultraviolet region, further augmenting the overall absorption intensity [54].

### 3.12. Endogenous Fluorescence Spectra

**Figure 9:**
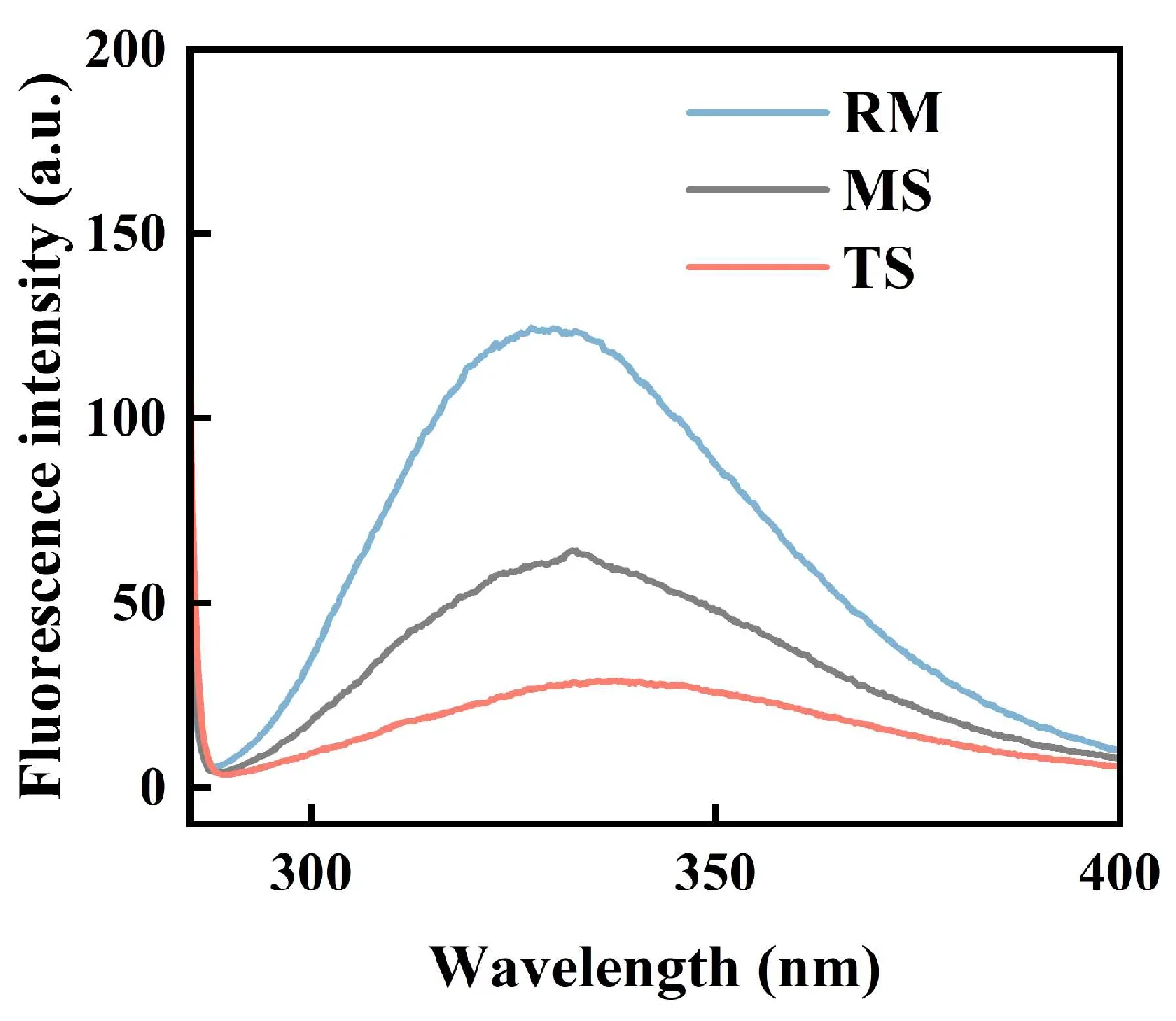
Endogenous fluorescence spectra of MPs extracted from crab meat subjected to different thermal treatments. RM, raw meat; MS, mild steaming; TS, traditional steaming.

The endogenous fluorescence spectra of MPs extracted from crab meat subjected to different thermal treatments are presented in Figure 9. With increasing thermal treatment intensity, the endogenous fluorescence intensity of the MPs decreased, accompanied by a red shift in the emission wavelength, suggesting that the microenvironment surrounding tryptophan residues became progressively more polar. These changes were more pronounced in the TS group than in the MS group.

The observed changes in endogenous fluorescence spectra are primarily attributed to heat-induced protein unfolding, which may expose tryptophan residues to the polar aqueous environment, leading to fluorescence quenching [55]. Fluorescence intensity decreased markedly in the TS group, accompanied by the most pronounced red shift, which indicates more extensive changes in the tertiary structure due to prolonged high-temperature exposure.

Interestingly, the UV spectra of the MS group were similar to those of the RM group, but the fluorescence intensity of the MS group was reduced compared with the RM group, and the emission wavelength shifted to a longer wavelength. This difference may be due to the fact that UV spectra are affected by the overall exposure of aromatic amino acid residues, while fluorescence spectra are sensitive to local environmental changes around tryptophan residues [56]. In the MS group, the overall exposure of aromatic amino acid residues was relatively low, but changes in the microenvironment of a small number of tryptophan residues could still be captured by fluorescence spectroscopy.

### 3.13. CD Spectra

**Figure 10:**
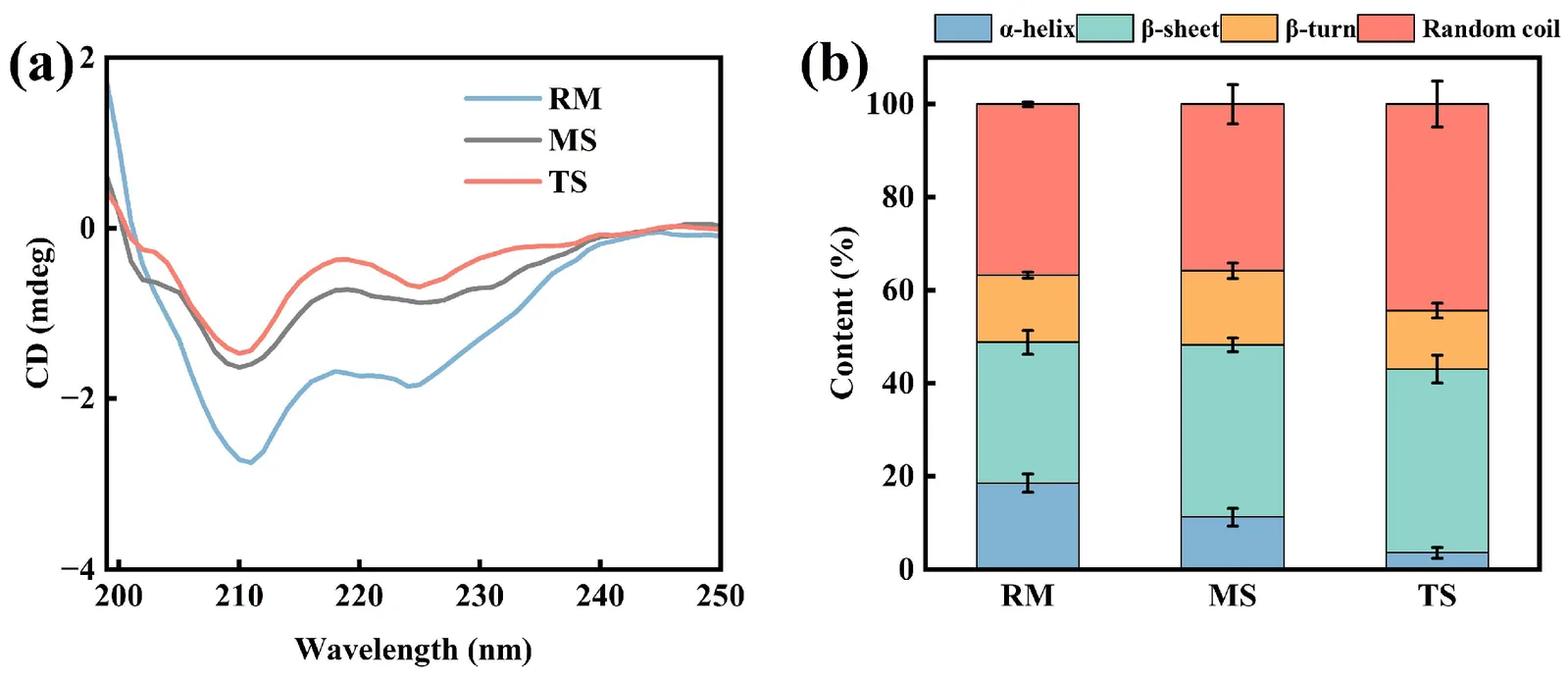
(**a**) Circular dichroism spectra and (**b**) secondary structure content of MPs extracted from crab meat subjected to different thermal treatments. RM, raw meat; MS, mild steaming; TS, traditional steaming.

CD spectroscopy is widely used for the characterization of protein secondary structure. The CD spectra of MPs in crab meat after different thermal treatments are shown in Figure 10a. The double-negative peaks at 210 nm and 224 nm are characteristic of α-helical structures, and the negative trough between 215–221 nm corresponds to β-sheet structures [57]. As the heating intensity increased, both the MS and TS groups exhibited a marked reduction in α-helix content, accompanied by a red shift in the 224 nm negative peak and notable changes in the broad trough between 215–221 nm, indicating that the secondary structure was altered. These alterations were more evident in the TS group.

The secondary structure composition of MPs in crab meat after different thermal treatments is presented in Figure 10b. The α-helix content decreased progressively from the RM group to the TS group (18.53 ± 1.96%, 11.23 ± 1.88%, and 3.60 ± 1.14%, respectively), whereas the β-sheet content increased in the same order (30.23 ± 2.55%, 37.03 ± 1.48%, and 39.43 ± 2.95%, respectively). The random coil content in the RM and MS groups was relatively similar (36.77 ± 0.49% and 35.83 ± 4.25%, respectively), but increased significantly in the TS group (44.43 ± 4.93%).

The reduction in α-helix content accompanied by an increase in β-sheet content suggests that thermal unfolding may promote a transition toward more ordered intermolecular β-sheet structures. In the MS group, short-term heating induced partial unfolding of α-helices. The unfolded polypeptide chains were likely to align with neighboring chains, promoting the formation of more energetically stable intermolecular β-sheet structures [36]. These changes were more prominent in the TS group. Prolonged exposure to high temperatures caused a significant loss of α-helices, and further unfolding and intermolecular aggregation may have contributed to the increased random-coil fraction. Thus, the secondary structure of proteins in the TS group became more disordered, particularly as indicated by the marked increase in the random-coil fraction [58].

Overall, the protein-level changes observed in the present study were consistent with previous studies, indicating that increasing thermal exposure progressively induced MP unfolding, denaturation, and aggregation, accompanied by changes in protein secondary structure and functional properties [38]. Protein denaturation and aggregation can disrupt the protein network responsible for water retention, alter the distribution and binding state of water within muscle, and consequently increase water loss during thermal processing [39]. Moreover, excessive protein aggregation and structural rearrangement may also affect the physicochemical and sensory properties of meat [37]. In the present study, the more pronounced protein structural changes in the TS group were accompanied by lower moisture content and higher centrifugal loss, as well as more pronounced changes in flavor and taste characteristics, suggesting that the deterioration of crab meat quality was associated with protein denaturation.

## 4. Conclusions

This study systematically evaluated the effects of TS and MS on the physicochemical quality, sensory characteristics, and MP properties of shelled Chinese mitten crab meat, with RM as the reference. Overall, the results demonstrated that MS provided a better balance between thermal treatment and quality preservation than TS. Compared with TS, MS caused less pronounced changes in physicochemical quality and moisture retention, while maintaining flavor and taste profiles closer to those of RM. At the protein level, the relatively limited changes in extractability, surface hydrophobicity, sulfhydryl groups, turbidity, particle size, fluorescence, and secondary structure indicated that MS resulted in less extensive protein unfolding, aggregation, and structural rearrangement. In contrast, TS promoted more pronounced protein conformational changes and aggregation, which were accompanied by greater losses in water retention and more evident changes in sensory characteristics. These findings suggest that MS reduced the shell–meat separation difficulty while minimizing heat-induced quality deterioration, providing a promising processing strategy for improving crab meat recovery while maximally preserving the inherent quality of crab meat.

However, several limitations should be acknowledged in the present study. The relatively low degree of thermal treatment of MS brought its quality characteristics closer to those of RM. Therefore, further investigations into microbial stability, storage properties, and shelf life are required to evaluate the practical applicability of MS-treated crab meat. In addition, the present study was conducted under laboratory-scale conditions, and further validation under industrial processing conditions is necessary to assess the feasibility and stability of MS for large-scale application.

## Data Availability

The data presented herein are available on request from the corresponding author. The data are not publicly available due to privacy restrictions.

## Supplementary Materials

The following supporting information can be downloaded at: https://www.mdpi.com/article/10.3390/foods15193540/s1.

## Abbreviations

The following abbreviations are used in this manuscript:

RM	Raw meat
MS	Part-specific mild steaming
TS	Traditional steaming
MP	Myofibrillar protein
TVB-N	Total volatile basic nitrogen
E-nose	Electronic nose
E-tongue	Electronic tongue
UV	Ultraviolet absorption
CD	Circular dichroism
